# The use of optical coherence tomography for the detection of ocular toxicity by ethambutol

**DOI:** 10.1371/journal.pone.0204655

**Published:** 2018-11-08

**Authors:** Brunella Maria Pavan Taffner, Felipe Berno Mattos, Mariana Cartaxo da Cunha, Fábio Petersen Saraiva

**Affiliations:** Department of Specialized Medicine, Division of Ophthalmology, Hospital das Clínicas of the Federal University of Espírito Santo, Vitória, Espírito Santo, Brazil; Charite Universitatsmedizin Berlin, GERMANY

## Abstract

**Objectives:**

To evaluate, through (OCT), alterations in retinal thickness, secondary to use of ethambutol in the treatment of patients with tuberculosis. In addition to studying the use of simpler semiological tools, such as Amsler and Ishihara, in the screening of these cases.

**Methods:**

Thirty patients with ethambutol were recruited from the reference service of tuberculosis treatment at the Federal University of Espírito Santo from May 2015 to July 2016. After clinical history, the following parameters were analyzed; best corrected visual acuity, biomicroscopy, tonometry, photomotor reflex testing, Ishihara test, Amsler’s grid test, color digital retinography and optical coherence tomography with CIRRUS HD-OCT (Humphrey-Zeiss) every 2 months during treatment with ethambutol. They were divided into two groups according to the treatment: (1) standard group, two months of ethambutol; (2) extended group, nine to twelve months of ethambutol.

**Results:**

There was a significant reduction in OCT thickness between the pre and post treatment times in ten eyes of the extended group, mean reduction of 7,8 microns and in seven eyes of the standard group, with an average of 5.57 microns. During the study, a significant reduction of retinal thickness was observed in both groups at two months of treatment, and the delta percentage was higher in those patients who presented reduction of visual acuity and / or change in the Ishihara test.

**Conclusion:**

There was a significant reduction in the thickness of the nerve fiber layer by OCT in the patients studied, being more pronounced in those submitted to the extended treatment regimen. This reduction was observed two months after the start of therapy, and was more significant in the cases that presented changes in the Ishihara test.

## Introduction

Tuberculosis (TB), caused by Mycobacterium tuberculosis, is a disease of high prevalence in our country. 9.6 million new cases of tuberculosis are registered per year, with Brazil among the 22 countries considered to have a high incidence of the disease, according to WHO [1]. We are facing an increase in the incidence of tuberculosis due to human immunodeficiency virus infection, immigration and multidrug resistance.

Ethambutol, associated with other drugs, has been widely used to treat infections caused by *Mycobacterium tuberculosis* and *Mycobacterium avium complex*. The adverse effects of this drug have been documented since it’s original use, the most severe being optic neuropathy, occurring in 22.5 cases per 1,000 patients [2]. It is a partially reversible toxic neuropathy, and the early discovery is fundamental to reduce permanent visual damage.

Exams that can detect early changes related to ocular toxicity by ethambutol are of extreme value for a better prognosis in this disease.

Optical coherence tomography, also known as OCT, is a diagnostic method that allows high-resolution images of cross-sectional retinal structures to be used as a non-invasive and non-contact technology for their realization [3]. The CIRRUS HD- OCT 4000 (Humphrey-Zeiss, Dublin, CA) has a scanning speed of 27,000 A-Scan / sec, an axial resolution of 5 μm and a transverse scan of approximately 20 μm, and repeatability standard deviation to 2.5 μm [4].

The OCT principle is based on low coherence interferometry [5] and the changes are observed as the relative difference in reflectivity at the optical interface of the structures examined [3,6]. There is a good correlation between OCT images and the corresponding anatomical structure. This is due to the difference in reflectivity of the retinal structures [7,8].

The objective of this study was to evaluate, through OCT, possible alterations in retinal thickness, secondary to toxicity by using ethambutol in the treatment of patients with tuberculosis. In addition, we have studied the use of other semiological tools, such as Amsler and Ishihara, in the screening of these cases.

## Materials and methods

A total of 30 subjects using ethambutol were enrolled in a reference service for treatment of tuberculosis at the Hospital das Clínicas of the Federal University of Espírito Santo (UFES) from May 2015 to July 2016. The research began after approval of the Research Ethics Committee of HUCAM / UFES on 05/26/15, CAAE 45217815.3.0000.5071.

Anamnesis were performed of all the patients including questioning of habits of life, comorbidities, medications in use and history of color blindness. The definition of the mycobacterium species was performed by sputum culture or biopsy material from the primary infectious site performed by the pulmonology department.

After a detailed clinical history, the participants were submitted to ophthalmologic examination consisted of: corrected visual acuity by the ETDRS system, biomicroscopy, tonometry, photomotor reflex test, relative afferent pupillary defect screening, color vision by the Ishihara test, Amsler grid test, and color digital retinography for recording the posterior pole. OCT was performed on the CIRRUS HD-OCT device (Humphrey-Zeiss, Dublin, CA) macular thickness protocols using 512 x 128 μm and nerve fiber layer (RNFL). Among the test results, only those with a signal intensity above seven were accepted. Total thickness, thickness per quadrant and thickness per hour were measured. Renal function was also assessed by measuring creatinine clearance.

Patients were examined every two months serially from the first month of treatment with ethambutol. The follow-up occurred until the following month after discontinuation of the medication.

All participants signed an informed consent form in agreement with the HUCAM/ UFES Ethics Committee, following the recommendations of the Declaration of Helsinki.

The inclusion criteria were: (1) absence of history of optic neuropathy (such as glaucoma, optic neuritis, etc), (2) absence of congenital dyschromatosis (such as color blindness) (3) absence of history of retinal diseases; (4) there is no opacity of the means in the slit lamp or fundoscopic examination; (5) no history of vision abnormalities or visual field defect; (6) no history of anterior segment diseases (except dry eye syndrome); (7) no history of drug use inducing ocular toxicity (except ethambutol); (8) no ocular tuberculosis or neurotuberculosis; (9) age greater than 18 years.

The 30 subjects were divided into two groups according to the treatment schedule indicated by the pneumology service: (1) standard treatment group, two months of ethambutol; (2) extended treatment group, nine to twelve months of ethambutol. It is known that the standard treatment for Micobacterium tuberculosis disease recommended by the Brazilian Ministry of Health is two months of rifampicin, isoniazid, pyrazinamide, and ethambutol, followed by four months of rifampicin and isoniazid [9]. The extended treatment is individualized depending on the mycobacterium isolated in the culture and the resistance of the same, and all patients with other mycobacteria, other than Micobacterium tuberculosis, have been treated for at least 12 months. Only continuous and prolonged use of ethambutol in the extended group were considered in the study. This standard and extended division into two groups was carried out to evaluate whether prolonged use of ethambutol could lead to a greater number or severity of ophthalmologic changes.

Patients developed signs or symptoms of toxic optic neuropathy were referred to the prescriber of ethambutol immediately for discontinuation of the medication.

Initially all variables were analyzed descriptively. For the quantitative variables, this analysis was done by observing the minimum and maximum values, and the calculation of means, standard deviations and median. Absolute and relative frequencies were calculated for the qualitative variables.

A separate analysis was performed between right and left eye for each of the parameters studied, to avoid the problem of correlating equivalent areas of right and left eye retina.

For the comparison of means of two groups, the Student’s t-test was used, when the normality assumption of the data was rejected, the non-parametric Mann-Whitney test was used.

To test the homogeneity between proportions, the chi-square test or Fisher’s exact test was used.

For the comparison of the groups throughout the evaluations was used the Analysis of Variance with repeated measures.

When the normality assumption of the data was rejected, Wilcoxon’s non-parametric test was used to compare the pre and post moments in the quantitative data. For the qualitative data the non-parametric McNemar test was used.

The software used for the analysis was SPSS 17.0 for Windows.

The level of significance used for the tests was 5%.

## Results

The 30 subjects were examined continuously during ethambutol use. Four patients lost follow-up during the study. The remaining 26 individuals were divided into two groups: twelve patients in the standard group (seven in the female and five in the male); and fourteen patients in the extended group (six females and eight males), according to the treatment defined by the pulmonology department.

Patients did not have any specific ocular symptoms on the first examination and baseline visual function tests were within normal range.

The mean age was 43.5 years (Standard Deviation 14,399) and the mean weight was 63.338 kg (Standard Deviation 6,980).

As for living habits, eight people reported alcoholism, seven smoking, and eight use of illicit drugs. In the ethnic category, fourteen considered themselves white, nine brown and three black. As for comorbidities, two had diabetes mellitus and three systemic arterial hypertension. Of all the evaluated, three individuals are carriers of HIV, all in an extended treatment regime. These variables did not differ significantly between the groups.

Twenty cases with *Micobacterium tuberculosis*, four *Micobacterium kansasii* and two *Micobacterium avium* were analyzed. In the statistical analysis, the groups showed a significant difference in relation to mycobacteria (p = 0.030). Standard treatment presented a higher percentage of cases with Micobacterium tuberculosis when compared to the extended treatment, as detailed in Table 1.

**Table 1:** Patients on ethambutol therapy: Standard treatment group and extended treatment group.

Patient	Treatment	Age	Sex	Ethnicity	Mycobacteria	Location
**1**	Extended	65	M	White	Avium	Pulmonary
**2**	Extended	54	F	Black	Avium	Pulmonary
**3**	Extended	38	F	Brown	Tuberculosis	Pulmonary
**4**	Extended	65	F	White	Kansasii	Pulmonary
**5**	Extended	46	M	White	Tuberculosis	Pulmonary
**6**	Extended	41	F	White	Tuberculosis	Pulmonary
**7**	Extended	32	M	White	Kansasii	Pulmonary
**8**	Extended	37	M	Black	Tuberculosis	Pulmonary
**9**	Extended	68	F	White	Kansasii	Pulmonary
**10**	Extended	46	F	Brown	Tuberculosis	Pulmonary
**11**	Extended	25	M	White	Tuberculosis	Pulmonary
**12**	Extended	34	M	White	Kansasii	Pulmonary
**13**	Extended	43	M	Brown	Tuberculosis	Pulmonary
**14**	Extended	74	M	White	Kansasii	Pulmonary
**15**	Standard	38	F	White	Tuberculosis	Cutaneous
**16**	Standard	58	F	White	Tuberculosis	Cutaneous
**17**	Standard	34	F	Brown	Tuberculosis	Cutaneous
**18**	Standard	36	F	Brown	Tuberculosis	Pleural
**19**	Standard	60	M	Brown	Tuberculosis	Pulmonary
**20**	Standard	49	F	White	Tuberculosis	Pulmonary
**21**	Standard	32	F	White	Tuberculosis	Pleural
**22**	Standard	37	M	White	Tuberculosis	Ganglionary
**23**	Standard	24	F	Black	Tuberculosis	Pulmonary
**24**	Standard	23	M	Brown	Tuberculosis	Pulmonary
**25**	Standard	50	M	Brown	Tuberculosis	Pulmonary
**26**	Standard	22	M	White	Tuberculosis	Pulmonary

Regarding the location of the Micobacterium infection, twenty cases were pulmonary, two pleural, three cutaneous and one lymph node. From Fisher’s exact test, we observed that the groups showed a significant difference in relation to the infection location (p = 0.004). The standard group presented a higher percentage of cases with cutaneous, lymph node and pleural tuberculosis when compared to the extended group.

There was no statistically significant difference between the groups regarding the weight and age variables.

The creatinine clearance of all patients at the start of treatment was within the normal range, with no statistical difference between the groups. Similarly, the intraocular pressure was maintained during all the research within the range considered normal.

All participants presented negative Ishihara test in the first evaluation.

The Amsler grid did not present a significant change in the analysis between the groups, and between the pre and post treatment.

In both groups there was a significant decrease in the temporal parafoveolar macular thickness of the right eye after the start of treatment (p = 0.005). In the other quadrants we did not observe significant changes to the OCT. The same change was not significant in the left eye.

In the analysis of the nerve fiber layer of the optic disc, we observed a significant decrease in the right eye nasal quadrant in both groups from the initial moment to the end of the analysis (p = 0.017). No other changes in the quadrants of both eyes.

In the hour chart of the right eye, a significant reduction of the nerve fiber layer was observed at 3 hours in both groups (p = 0.002).

In the 5-hour analysis, the groups showed a significant difference in behavior throughout the evaluations (p = 0.03), with the group extended with a significant decrease from the initial moment to the final moment (p = 0.031).

In the left eye, in the analysis of the hour chart, a significant reduction was observed at 8 hours in both groups studied after the use of ethambutol (p = 0.037). Without other statistically significant differences between the groups in the hour chart. Tables with all raw data are available in attachments.

Knowing that the standard deviation of the repeatability of the device is 2.5 μm [4], there was a relevant reduction in OCT thickness between the pre and post treatment times in ten eyes of the extended group, with a mean of 7,8 μm (between 3 and 19) and; in seven eyes of the standard group, with a mean of 5.57 μm (variation between 3 and 10).

**Fig 1:**
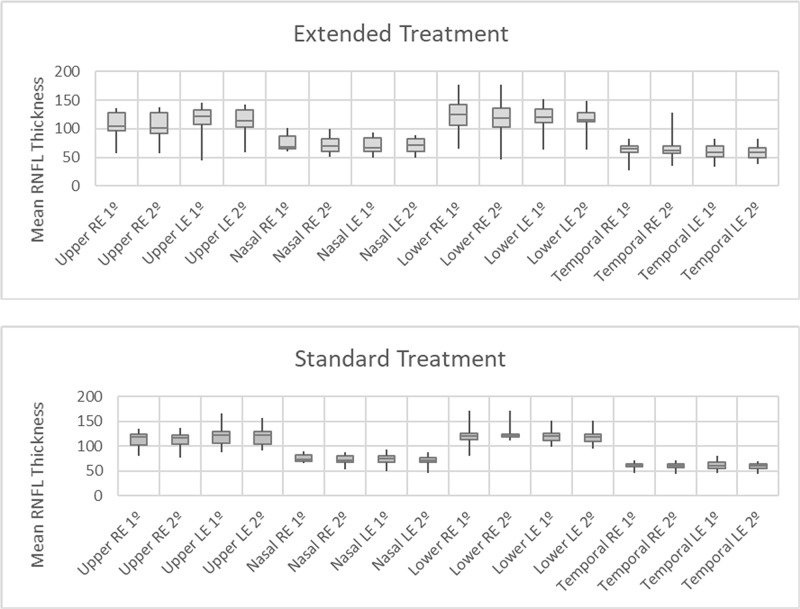
RNFL thickness, measured in micrometers, standard versus extended group, before start medication (first exam) and after two months of treatment (second exam). RNFL for each patient profile is measured in quadrants (temporal, upper, nasal, lower). In the second treatment evaluation, at two months, there are cases with reduction of the retinal thickness, in both groups. RE: Right Eye; LE: Left Eye.

During the study, it can be observed that in the second treatment evaluation, at two months, there are cases with reduction of the retinal thickness, as can be seen in Fig 1.

There was a decrease in OCT thickness of the optic disc (greater than 2.5 microns) between the initial moments and 2 months of treatment in ten eyes of the extended group, with a mean of 9.4 μm (variation between 3 and 21) and; in seven eyes of the standard group, with an average of 5.57 μm (variation between 3 and 10). No statistically significant difference between groups in relation to the delta percentage of variation between initial care and 2 months of treatment and between pre and post treatment.

In the extended group, throughout the treatment, three presented reduction of at least one line of sight in the measurement of visual acuity, besides, also, of alteration in the test of Ishihara. Two cases presented change only in the Ishihara test, with no loss of sight lines. None of these changes were observed in the standard group (p = 0.043).

Patients with reduced visual acuity and / or change in the Ishihara test had a greater delta percentage decrease when compared to the other patients, in the optic disc OCT lower thickness of the right eye (p = 0.046), the 5-hour OCT of the right eye (p = 0.003), the 7-hour OCT left eye (p = 0.001) when comparing the initial care and the 2-month care.

Patients with reduced visual acuity and / or change in the Ishihara test presented a greater delta percentage decrease between the first and second care compared to the other patients in the macula OCT thickness of the upper parafoveolar right eye (p = 0.043), nasal parafoveolar (p = 0.009), upper perifoveolar (p = 0.044), nasal perifoveolar (p = 0.005), lower perifoveolar (p = 0.001); in the left eye upper parafoveolar (p = 0.029), nasal parafoveolar (p = 0.038), lower perifoveolar (p = 0.01). The same comparison did not present statistical significance when comparing pre and post treatment.

After discontinuation of ethambutol, there was complete recovery of vision in three of the five cases. The other two had only partial recovery and developed optic disk pallor.

## Discussion

Since its introduction in 1961, ethambutol has been used in the treatment of tuberculosis. The World Health Organization Guideline 2009 recommends the addition of ethambutol throughout the standardized treatment of new cases of active TB in populations with increased prevalence of resistance to isoniazid to reduce the risk of multiple drug resistant strains. Brazil is included in this recommendation. [1]

The side effects of ethambutol were first described in 1962 by Carr and Henkind [10]. Leibold reported in 1966 that the ocular toxicity of the drug would not be a cause of concern when given at low dosages (below 25 mg / kg per day) and for short periods of time [11] Recent studies show people receiving 25 mg / kg per day had to 5% to 6% incidence of optic neuropathy, and the incidence of optic neuropathy at doses of 15 mg / kg per day was reportedly less than 1% [12,13]. It is noteworthy that most of these reports are from tertiary references centers and detection bias can be found because serious cases tend to be reported. Therefore, the true incidence of optic neuropathy may be unknown.

Current regimens recommend dosages of 15 mg / kg per day in patients who have not received prior antituberculosis therapy. If antituberculosis therapy was given before, initial treatment with 25 mg / kg per day for 60 days, followed by 15 mg / kg per day [14].

The presentation of the basic treatment scheme for tuberculosis according to the Ministry of Health of Brazil are tablets with fixed doses of 150mg of rifampicin, 75mg of isoniazid, 400mg of pyrazinamide and 275mg of ethambutol for adults and adolescents. For children under 10 years of age the combination of rifampicin, isoniazid and pyrazinamide remains. The amount of tablets administered per day is established according to the weight range: between 20 and 35 kg are 2 tablets per day; between 36 and 50 kg are 3 tablets per day and in greater of 50 kg are 4 tablets per day. In special schemes the dose of the tablet used is 400 mg of ethambutol administered according to the weight range: between 20 and 35 kg are 1 or 2 tablets per day; between 36 and 50 kg are 2 or 3 tablets a day and in greater than 50 kg are 3 tablets a day. In treatment regimens for multiresistance, the dosage is also defined according to the weight range: up to 20 kg is used 25mg / kg / day; between 21 and 35 kg are 400 to 800 mg daily; between 36 and 50 kg are 800 to 1200 mg / day and in greater of 50 kg are 1200 mg per day, as prescribed by the attending physician. [15]

It is emphasized that ethambutol is eliminated by the renal route both by glomerular filtration and by tubular secretion [16]. For this reason, any kidney disease could impact the patient’s ability to remove ethambutol from the serum. Patients with renal disease should have their dose adjusted based on their glomerular filtration rate. In this study, the serum creatinine dosage of all participants was within the normal range.

Due to an increase in the occurrence of multiresistant mycobacterial infections, the prescription of higher doses of ethambutol for long periods of time has become a necessity. This increases the risk of side effects of ethambutol [17].

Clinical features of ethambutol-induced optic neuropathy resemble those of a compressive optic neuropathy with retrobulbar neuritis in subacute clinical course with reduced contrast sensitivity, painless loss of central vision, cecocentral scotomas, and dyschromatopsia. Symptoms usually appear between four and twelve months after the onset of ethambutol, but rarely, they may also occur within a few days of initiation of therapy [18]. Fundoscopy is typically normal, especially in the early stages of the disease. Therefore, studies that evaluate the risk factors for the development of optic neuropathy due to the use of ethambutol, as well as the recognition of tests that can identify early signs of neuropathy, are important and necessary for medical practice.

Although the exact mechanism of this ocular neurotoxic effect is largely unknown, animal studies have demonstrated toxicity of ethambutol in rat retinal ganglion cells with vacuole formation in neurons and astrocytes following exposure to ethambutol in animal models [19].

There are authors who suggest that the toxic effects of ethambutol are reversible [20,21]. However, other studies have reported that, even after ethambutol stagnation, 40–50% of patients experienced permanent loss of visual acuity [22,23]. In this present analysis, it was observed that two cases (40%) did not completely recover vision after discontinuation of ethambutol and, in addition, developed optic disk pallor, characterizing permanent structural damage. The risk of definitive visual loss reinforces the need for early diagnosis of neuropathy and immediate interruption of medication.

Chromatopsia is reported as the first symptom of toxic optic neuropathy. Garg et al. observed color vision abnormalities in 12.6% of the eyes of 64 patients studied [24]. The Ishihara test was therefore used to observe loss of color sensitivity. It is worth mentioning that this test is considered the gold standard for the identification of congenital deficiencies for color vision, but it can also be used for acquired deficiencies [25–27]. In the present research, the positivity of this test was associated to the cases with greater reduction of the retinal thickness by the OCT and with all the patients that had reduced visual acuity.

The Farnsworth-Munsell test was the first indication for the detection of acquired color vision loss, but it was not chosen for the present study due to its lower availability and greater complexity in the accomplishment, which limits its use to medical ophthalmologists.

The Amsler grid proved irrelevant in this analysis, since there was no record of change in any of the participants.

A few papers have demonstrated the usefulness of optical coherence tomography (OCT) in the detection of retinal changes in patients with optic neuropathy associated with the use of ethambutol [28]. The prevailing hypothesis is that these changes cause papillomacular defects [28–30].

Most of the studies using OCT reported that the thickness of the NRT reduces with antituberculosis therapy [28, 29, 31, 32]. However, in contrast to such findings, Kyoung et al. reported an increase in the thickness of the nerve fiber layer during treatment with ethambutol [33].

Ethambutol optic neuropathy is generally bilateral and may be asymmetric. [34] This asymmetry was observed in OCT of both groups. Asymmetry of the thickness of the CFNR between right eye and left eye after treatment with ethambutol has already been demonstrated by Zoumalan, [29] and corroborates with the findings of this research.

In the present study, it was observed that both patients in standard treatment and in extended treatment presented a significant reduction in the thickness of the nerve fiber layer detected by OCT, being more pronounced in the individuals in the extended treatment group, even in patients without visual complaints and may indicate a possible subclinical neurotoxicity of long-term outcome yet unknown. It is interesting to note that it is possible to observe a reduction of the retinal thickness two months after the start of treatment, especially in those patients with reduced visual acuity and / or change in the Ishihara test. This raises the possibility that the earlier and more pronounced reduction of the nerve fiber layer is involved in the appearance of visual symptoms.

One of the limitations of the research was the fact that we could not have evaluated the use of ethambutol alone. Sahin et al. have already shown ocular toxicity due to the use of isoniazid in rats, however, there are no reports of this association in humans [35].

## Conclusion

There was a significant reduction in the thickness of the nerve fiber layer by OCT in the patients studied, being more pronounced in those submitted to the extended treatment regimen. This reduction was observed two months after the start of therapy, and was more significant in the cases that presented changes in the Ishihara test.

Most cases with reduction of retinal thickness by OCT did not present reduction of visual acuity or change in the Ishihara test.

Further studies are needed to elucidate the risk factors and intervals required between OCT screening tests for early signs of ethambutol optic neuropathy.

## Supporting information

S1 Table Optical coherence tomography data of all patients. (DOCX)

## References

[1] World Health Organization. Global tuberculosis report, 19ª edição, World Health Organization. 2014.

[2] Ezer N, Benedetti A, Darvish-Zargar M, Menzies D. Incidence of ethambutol-related visual impairment during treatment of active tuberculosis. Int J Tubercu Lung Dis. 2013 2; 17(4): 447–455. 10.5588/ijtld.11.0766 23394767

[3] Huang D, Swanson EA, Lin CP, Schuman JS, Stinson WG, Chang W, et al Optical coherence tomography. Science. 1991 22;254(5035): 178–81. 10.1126/science.1957169 PMC4638169 1957169

[4] Carl Zeiss Meditec, Inc. Manual do usuário do CIRRUS HD-OCT, Rev. A. 2015.

[5] Fercher AF, Mengedoht K, Werner W. Eye-length measurement by interferometry with partially coherence light. Opt Lett. 1988; 13: 186–8. 19742022 10.1364/ol.13.000186

[6] Hee MR, Puliafito CA, Wong C, Reichel E, Duker JS, Schuman JS, et al Optical coeherence tomography of the human retina. Arch Ophthalmol. 1995; 113(3):325–32. 7887846 10.1001/archopht.1995.01100030081025

[7] Puliafito CA, Hee MR, Lin CP, Reichel E, Schuman JS, Duker JS, et al Imaging of macular diseases with optical coherence tomography. Ophthalmology. 1995;102(2):217–29. 7862410 10.1016/s0161-6420(95)31032-9

[8] Toth CA, Narayan DG, Boppart SA, Hee MR, Fujimoto JG, Birngruber R, et al A comparison of retinal morphology viewed by optical coherence tomography and by light microscopy. Arch Ophthalmol. 1997;115(11):1425–8. Erratum in: Arch Ophthalmol 1998;116(1):77. 9366674 10.1001/archopht.1997.01100160595012

[9] Ministério da Saúde do Brasil. Secretaria em Vigilância em Saúde. Departamento de Vigilância Epidemiológica. Programa Nacional de Controle da Tuberculose. Nota técnica sobre as mudanças no tratamento da tuberculose no Brasil para adultos e adolescentes–Versão 2. Brasília: Ministério da Saúde. 2009.

[10] Ronald E. Carr M.D.; Paul Henkind M.D. Ocular manifestations of ethambutol toxic amblyopia after administration of an experimental antituberculous drug. Arch Ophthalmol. 1962; 67(5): 566–571. 13876814 10.1001/archopht.1962.00960020566009

[11] Leibold JE. The ocular toxicity of ethambutol and its relation to dose. Ann NY Acad Sci. 1966; 135:904–9. 5220245 10.1111/j.1749-6632.1966.tb45532.x

[12] Sivakumaran P, Harrison AC, Marschner J, et al Ocular toxicity from ethambutol: a review of four cases and recommended precautions. N Z Med J 1998; 111: 428–30. 9861923

[13] Petri WA Jr. Drugs used in the chemotherapy of tuberculosis, *Mycobacterium avium* complex disease, and leprosy In: Hardman JS, Limbird LE, eds. *Goodman & Gilman’s The Pharmacological Basis of Therapeutics*. 10th ed New York, NY: McGraw-Hill; 2001:1279–1280.

[14] Mosby’s DrugConsult 2002. St Louis, Mosby. 2002.

[15] Manual de recomendações para o controle da tuberculose no Brasil/ Ministério da Saúde, Secretaria de Vigilância em Saúde, Departamento de Vigilância Epidemiológica.—Brasília: Ministério da Saúde, 2011.

[16] Petri WA Jr. Goodman & Gilman’s the pharmacological basis of therapeutics, 2006; 11: 47–4.

[17] Alvarez K. L., & Krop L. C. Ethambutol-induced ocular toxicity revisited. Annuals in Pharmacotherapy, 1993 27; 102–103. 10.1177/106002809302700126 8431610

[18] Schild H Fox. Rapid-onset reversible ocular toxicity from ethambutol therapy. Am J Med. 1991; 90: 404–6. 1848398

[19] Yoo YH, Jung KH, Sadun AA, et al Ethambutol-induced vacuolar changes and neuronal loss in rat retinal cell culture: mediation by endogenous zinc. Toxicol Appl Pharmacol 2000; 162: 107–114. 10.1006/taap.1999.8846 10637134

[20] Chen L, Liang Y. Optic nerve neuropathy by ethambutol toxicity. Zhonghua Jie He He Hu Xi Za Zhi. 1999 5; 22(5): 302–4. 11775861

[21] Woung LC, Jou JR, Liaw SL. Visual function in recovered ethambutol optic neuropathy. J Ocul Pharmacol Ther, 1995, 11(3): 411–419. 10.1089/jop.1995.11.411 8590273

[22] Kumar A., Sandramouli S., Verma L., Tewari H. K., & Khosla P. K. Ocular ethambutol toxocity: is it reversible? Journal of Clinical Neuroophthamology, 1993, 13, 15–17. 8501256

[23] Tsai RK, Lee YH. Reversibility of ethambutol optic neuropathy. J Ocul Pharmacol Ther 1997; 13: 473–7. 10.1089/jop.1997.13.473 9326729

[24] Garg P, Garg R, Prasad R, Mishra AK. A prospective study of ocular toxicity in patients receiving ethambutol as a part of directly observed treatment strategy therapy. Lung India 2015; 32:16–9. 10.4103/0970-2113.148428 25624590 PMC4298911

[25] Birch J. Efficiency of the Ishihara test for identifying red-green colour deficiency. Ophthalmic Physiol Opt. 1997; 17(5): 403–8. 9390366

[26] Crone RA. Quantitative diagnosis of defective colour vision. A comparative evaluation of the Ishihara test, the Farnsworth Dichotomous test and the H-R-R polychromatic plates. Am J Ophthalmol. 1961; 51: 298–305. 13696552

[27] Hardy LH, Rand G, Rittler MC. Tests for deteccion of colour blindness. An evaluation of the Ishihara test. AMA Arch Ophthalmol. 1945; 3534: 295–302. 10.1001/archopht.1945.00890190297005 21009152

[28] Chai SJ, Foroozan RD. Decreased retinal nerve fibre layer thickness detected by optical coherence tomography in patients with ethambutol induced optic neuropathy. Br J Ophthalmol. 2007 7; 91(7): 895–7. 10.1136/bjo.2006.113118 17215265 PMC1955652

[29] Zoumalan CI, Agarwal M, Sadun AA. Optical coherence tomography can measure axonal loss in patients with ethambutol induced optic neuropathy. Graefes Arch Clin Exp Ophthalmol 2005; 243: 410–416. 10.1007/s00417-004-1053-1 15565293

[30] Menon V, Jain D, Saxena R, Sood R. Prospective evaluation of visual function for early detection of ethambutol toxicity. Br J Ophthalmol 2009; 93: 1251–1254. 10.1136/bjo.2008.148502 19525243

[31] Zoumalan CI, Sadun AA. Optical coherence tomography can monitor reversible nerve-fibre layer changes in a patient with ethambutol-induced optic neuropathy. Br J Ophthalmol 2007; 91: 839–840. 10.1136/bjo.2006.107326 17510481 PMC1955581

[32] Kim BK, Ahn M. The use of optical coherence tomography inpatients with ethambutol-induced optic neuropathy. J Korean Ophthalmol Soc 2010; 51: 1107–1112.

[33] Kim KyoungLae & Park Sung Pyo: Visual function test for early detection of ethambutol induced ocular toxicity at the subclinical level, Cutan Ocul Toxicol, 2015; 35; 3: 228–232. 10.3109/15569527.2015.1079784 26361935

[34] Fraunfelder FT, Fraunfelder FW: *Drug-Induced Ocular Side Effects* (5th edn), Fraunfelder FT, Fraunfelder FW (Eds), Butterworth-Heinemann, Woburn, Massachusetts, USA (2001):824.

[35] Şahin A, KürşatCingü A, Kaya S, Türkcü G, Arı Ş, Evliyaoğlu O, et al The protective effects of caffeic acidphenethylester in isoniazid and ethambutol-induced ocular toxicity of rats. Cutan Ocul Toxicol 2013; 32:228–23. 10.3109/15569527.2012.759958 23351037

