# Supplementary material for: The use of optical coherence tomography for the detection of ocular toxicity by ethambutol
Source: PLoS One. 2018 Nov 8;13(11):e0204655. doi: 10.1371/journal.pone.0204655 (PMC6224029; doi:10.1371/journal.pone.0204655)
Supplement: S1 Table — (DOCX) [file pone.0204655.s001.docx]

**S1 Table. Optical coherence tomography data of all patients.**

**S1 Table A. Data of optical coherence tomography of retina nerve fibers layer (RNFL) of right eye and left eye, measured in micrometers.**


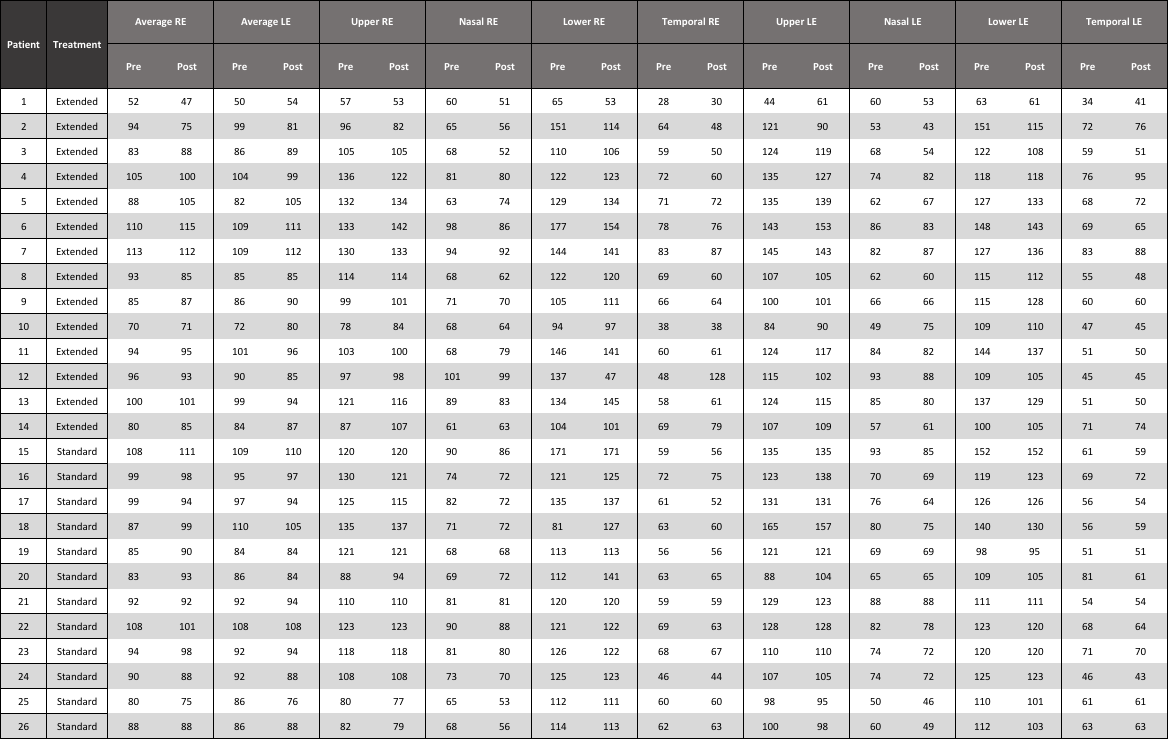


LE: Left Eye; RE: Right Eye.

**S1 Table B. Data of optical coherence tomography of retina nerve fibers layer (RNFL) of right eye, time chart, measured in micrometers.**


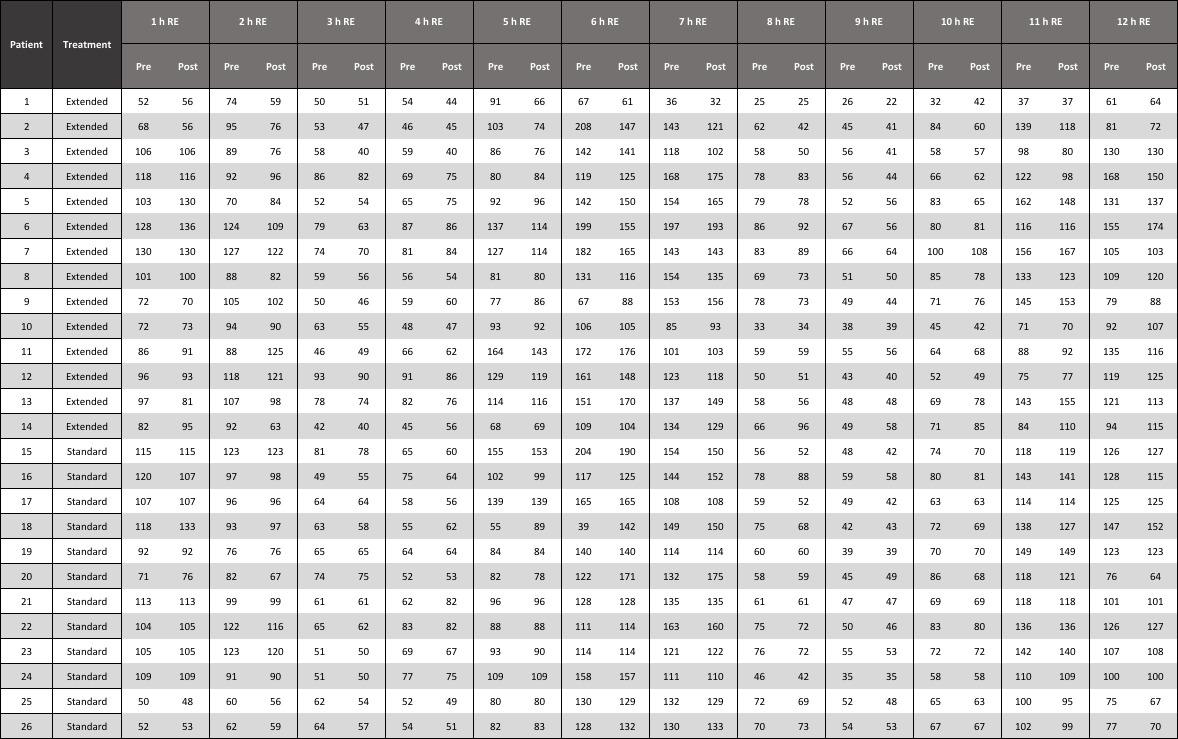


RE: Right Eye.

**S1 Table C. Data of optical coherence tomography of retina nerve fibers layer (RNFL) of left eye, time chart, , measured in micrometers.**


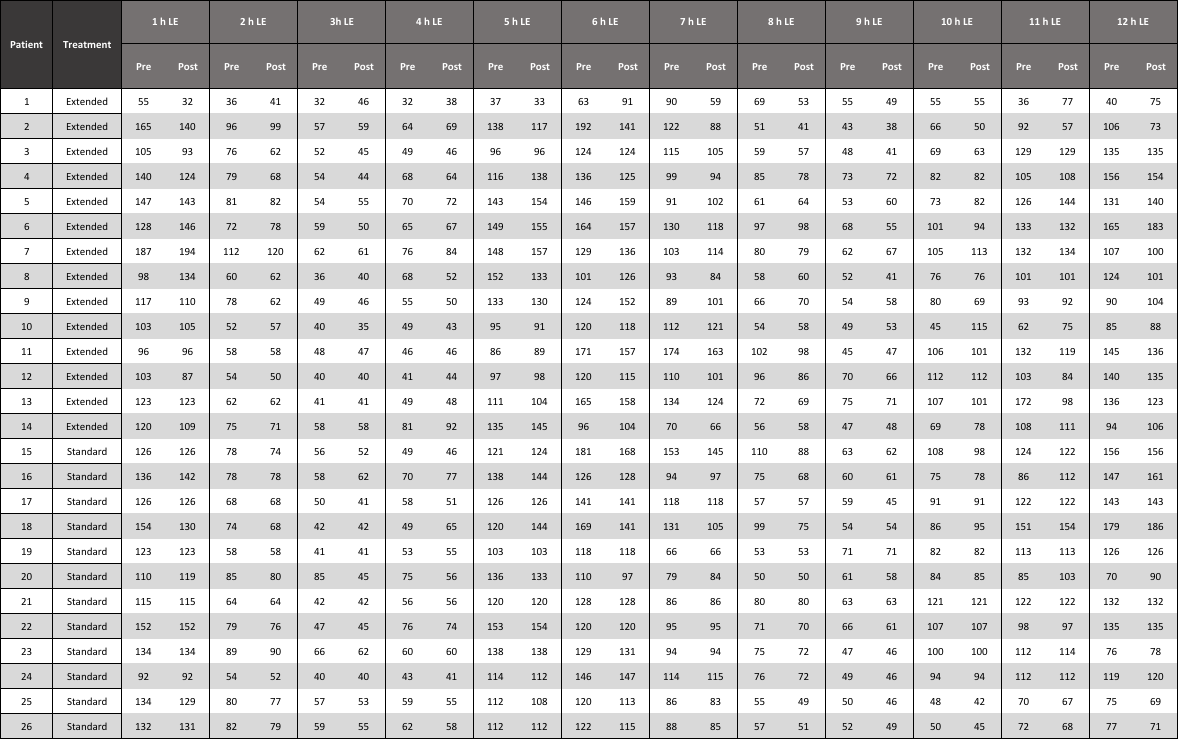


LE: Left Eye.

**S1 Table D. Data of optical coherence tomography of macular thickness analysis of the right eye, measured in micrometers.**


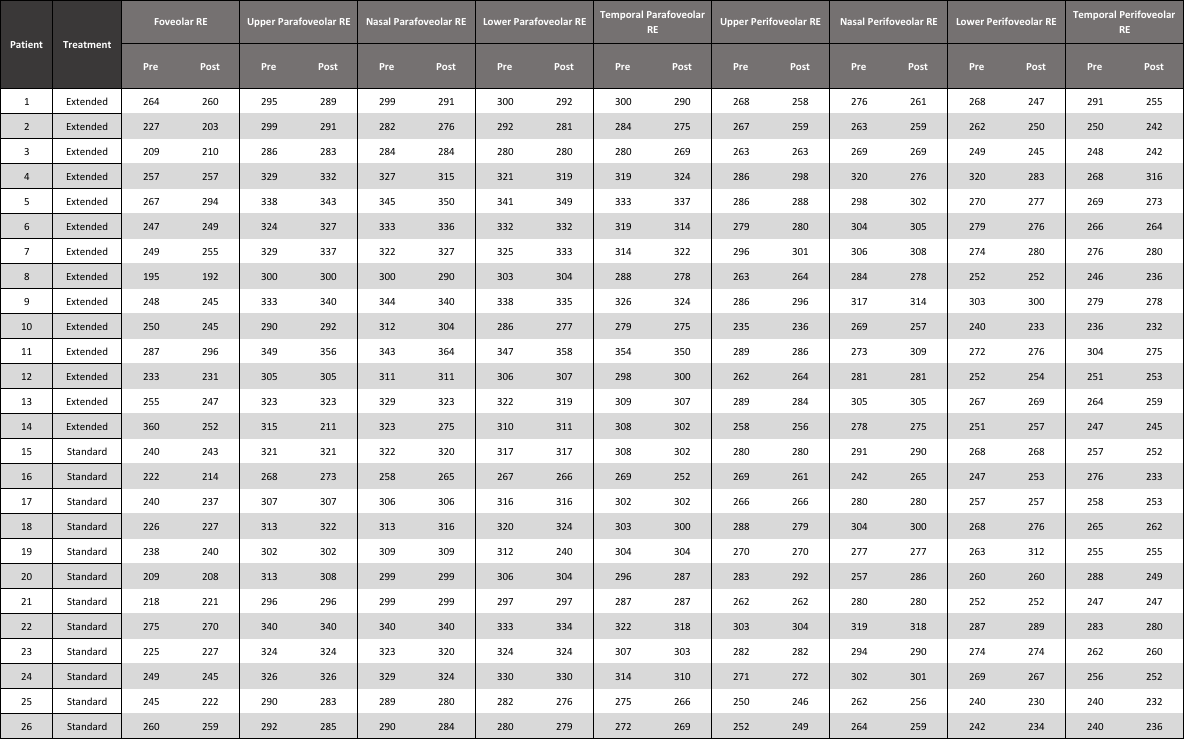


RE: Right eye.

**S1 Table E. Data of optical coherence tomography of macular thickness analysis of the left eye, measured in micrometers.**


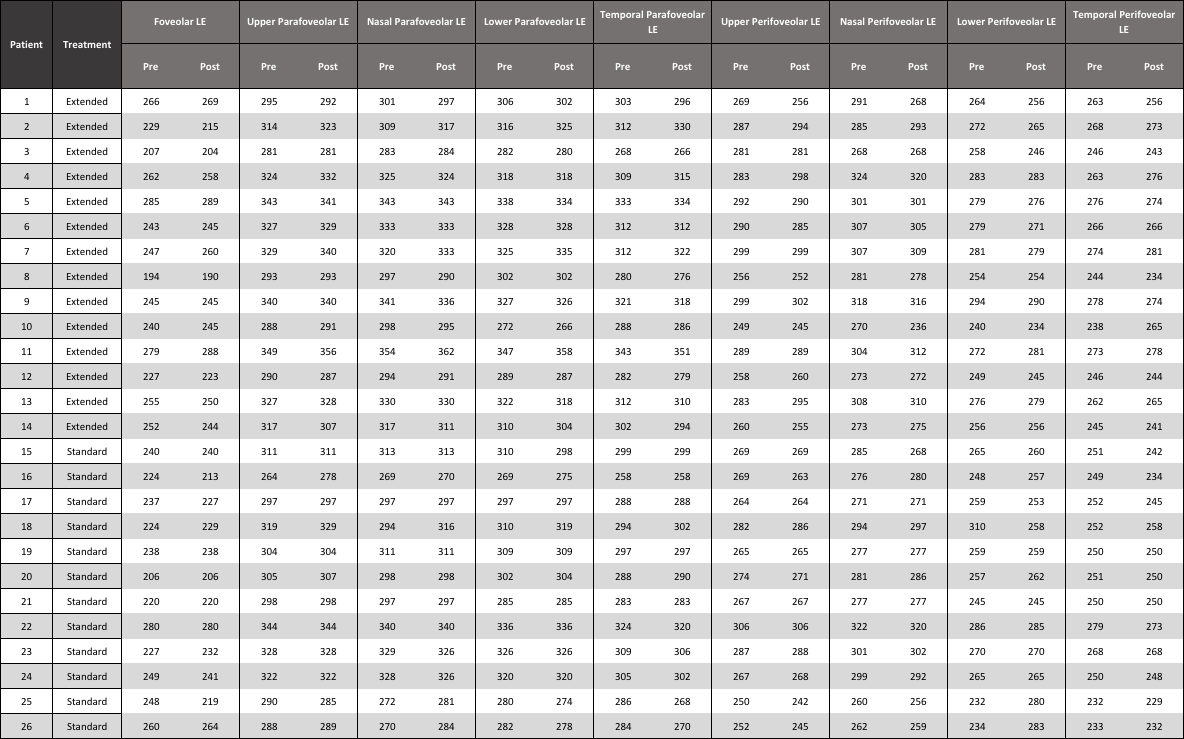


LE: Left Eye
